# A Comprehensive Review of Progress in Preventing Urinary Infections Associated with the Use of Urinary Catheters: A Dual Analysis of Publications and Patents

**DOI:** 10.3390/idr17030064

**Published:** 2025-06-04

**Authors:** Brunella Corrado, Aniello Cammarano, Stefania Dello Iacono, Emilia Renzi, Rosalba Moretta, Maria Emilia Mercurio, Laura Ascione, Annunziata Cummaro, Caterina Meglio, Luigi Nicolais

**Affiliations:** 1 Materias Srl, Corso Nicolangelo Protopisani 50, 80146 Naples, Italy; 2 Institute of Polymers, Composites and Biomaterials (IPCB), National Research Council, Piazzale Enrico Fermi 1, 80055 Portici, Italy; 3 Sanidrink Srl, Corso Nicolangelo Protopisani 50, 80146 Naples, Italy; 4 Department of Chemical, Materials and Production Engineering, University of Naples Federico II, Piazzale Vincenzo Tecchio, 80125 Naples, Italy

**Keywords:** CAUTIs, bibliometric analysis, patent landscape analysis, network map, VOS analysis, PRISMA

## Abstract

Catheter-associated urinary tract infections (CAUTIs), caused by microbial colonization of catheter surfaces, are among the most common healthcare-associated infections and significantly strain healthcare systems worldwide. This review aimed to provide a comprehensive analysis of the current scientific literature and the patent landscape from 2014 to 2024 on strategies for preventing CAUTIs. A systematic search was conducted using the PRISMA method, which involved searching the Scopus database for scientific evidence and analyzing patent search results on The Lens.org platform. Co-authorship and co-occurrence analysis unveiled key contributors and emerging themes within the scientific community. Simultaneously, an in-depth inspection of patents filed elucidated top origins, applicants, and classifications. Additionally, network analysis based on keywords from papers and patents revealed the scientific scenario and the innovation trends, enriching the understanding of technological advancements. It highlights emerging technologies, key actors, and potential gaps, providing valuable insights for researchers, clinicians, and industry stakeholders, thereby contributing to overcoming barriers to treating CAUTIs.

## 1. Introduction

**Figure 1:**
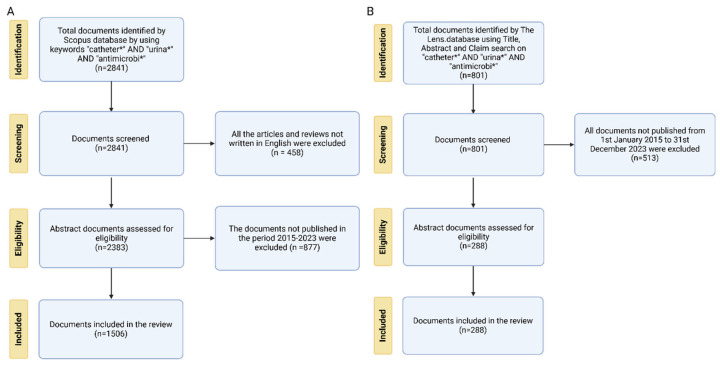
The PRISMA flow chart. (**A**) PRISMA flow diagram of bibliometric database analysis; (**B**) PRISMA flow diagram of patent database analysis.

Urinary catheters (UCs) are flexible tubes designed to be inserted through the urethra into patients’ bladder, allowing urine collection into a drainage bag [1]. Based on dwell time, UCs can be either intermittent (short-term) or indwelling (long-term). UCs are essential before and after specific surgeries or in case of impaired bladder function, when a safety system is required to drain, collect, and check urine. The catheter insertion process, especially for indwelling catheters, is an invasive procedure that has to be performed by specialized and trained healthcare personnel [2]. The medical and clinical staff must follow standard protocols for procedures, which involve hand washing, use of sterile gloves, and no-touch insertion methods, to ensure the asepticity of all necessary medical equipment [2]. Despite following these strict protocols, the risk of infections related to the use of catheters remains high [3]. Catheter-associated urinary tract infections (CAUTIs) occur when microorganisms colonize the catheter’s intraluminal surface or the extraluminal surface. The intraluminal colonization often results from contamination of the drainage system [4,5]. In contrast, extraluminal colonization typically occurs via bacterial migration from the urethral meatus along the external catheter surface, as described by Maki et al. [6] Both pathways provide a potential route for bacteria to enter the urinary tract, leading to infections. CAUTIs are among the most common nosocomial infections, accounting for approximately one million cases annually in both the United States and Europe. These infections and chronic wounds caused by prolonged bed rest [7] significantly burden the healthcare system [8]. Therefore, this bibliometric and patent analysis explores antimicrobial strategies for preventing CAUTIs, providing insights into both the scientific and patent landscape, according to a PRISMA method (Figure 1).

**Figure 2:**
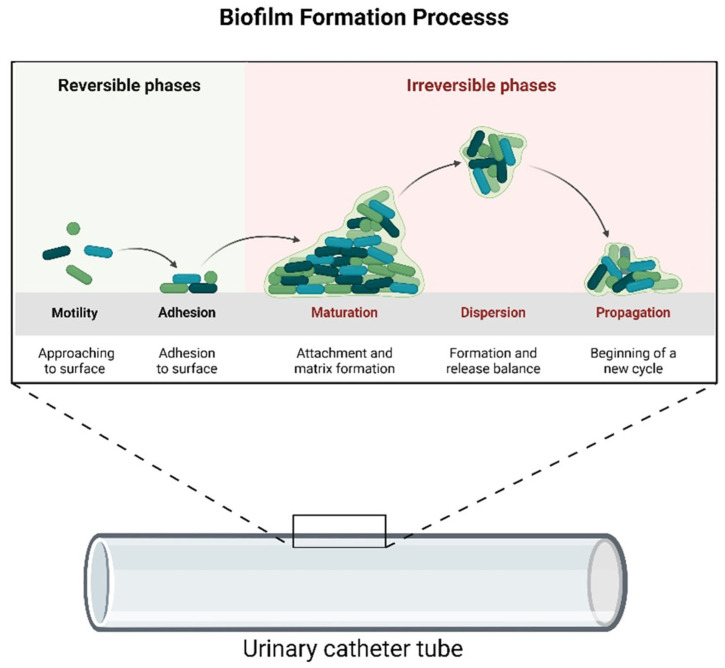
The biofilm formation process and maturation onto the catheter surface (image created with BioRender).

CAUTIs can cause several complications, such as orchitis, epididymitis, and prostatitis in males, whereas pyelonephritis, cystitis, and meningitis can occur independently of gender [9]. In addition to causing localized urinary tract infections, CAUTIs are a major risk factor for developing secondary bloodstream infections, including bacteremia and sepsis [10]. The progression from a localized infection to a systemic response significantly increases morbidity, mortality, and healthcare costs associated with catheter use. Like other hospital-acquired infections (HAIs), CAUTIs impose a substantial economic burden and contribute to significant mortality rates. The incidence of CAUTIs is noteworthy, contributing to increased medical complications, prolonged hospital stays, and healthcare costs [11,12,13]. Interestingly, the costs allocated for controlling and preventing HAIs are considerably lower than the expenses related to treating CAUTIs [14,15]. Among the risk factors associated with the development of CAUTIs [16,17], the duration of the catheterization [18], improper insertion or poor maintenance practices, gender differences [19,20], underlying health conditions [21], and age-related factors [22] are the most addressed. CAUTIs are caused by various microorganisms, including Gram-positive and Gram-negative bacteria and fungi [23,24]. The most common pathogens involved in CAUTIs are *Escherichia coli*, *Enterococcus* spp., *Klebsiella pneumoniae*, *Staphylococcus aureus*, and *Pseudomonas aeruginosa*, which can form a biofilm on the surfaces of UCs, contributing to persistent infections resistant to antibiotic therapy [25]. Biofilms are structured colonies of microorganisms that adhere to surfaces, forming an adhesive complex of a self-produced extracellular polymeric substance (EPS) [26]. Biofilms also act as a barrier that limits the penetration of antibiotics, contributing to antibiotic resistance [24]. Typically, the development of biofilms on catheters progresses through several distinct stages [27,28]. The initial stage involves the reversible attachment of bacteria on the catheter surface through the establishment of electrostatic or van der Waals interactions [29]. In the second stage, bacteria secure their attachment using cell adhesion structures like pili [30]. During stage three, the microorganisms start dividing and forming colonies, and cell communication occurs through quorum sensing (QS) [31]. In the fourth stage, microorganisms within the biofilm undergo phenotypic changes, adapting to the biofilm lifestyle. In the final stage, some microorganisms experience dispersion, releasing cells into the surrounding environment [32], then the dispersed cells can colonize new surfaces or contribute to infections at distant sites [32,33]. Figure 2 reports the biofilm formation stages on the UC surface.

**Figure 3:**
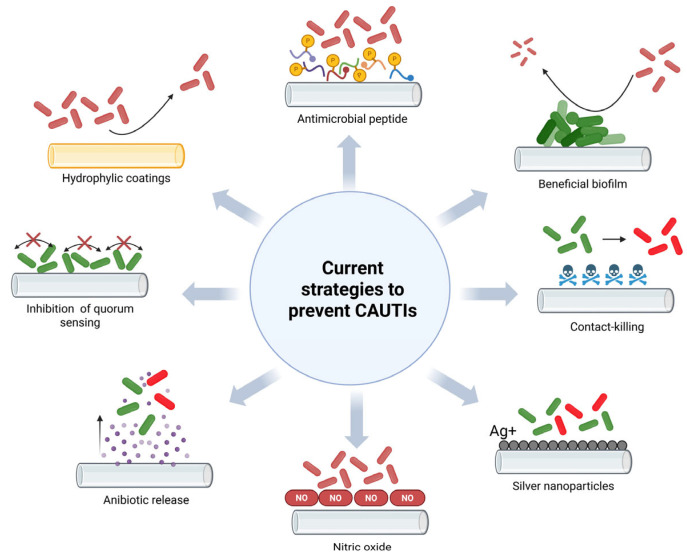
Antimicrobial strategies to prevent biofilm formation on the urinary catheter surface (image created with BioRender).

To prevent and reduce biofilm formation on the surface of catheter tubes, various strategies have been explored, encompassing coating and releasing of bactericidal agents [34], anti-biofouling tactics [35,36], biofilm destruction [37], bacterial interference [38], and functionalization of the inner/outer surface of catheters with antimicrobial peptides (AMPs) [39,40]. Current antimicrobial strategies are schematized in Figure 3.

The use of bactericidal substances can benefit from silver coating [34], both in the form of silver oxide and silver alloy [36,41,42], for its well-known antimicrobial properties [43]. Silver-coated catheters typically release silver ions, the active agents responsible for the antimicrobial effect, into the surrounding environment [44]. Indeed, silver ions interfere with the bacterial cell membranes, enzymes, and DNA, disrupting vital cellular functions [41]. In recent times, silver nanoparticles (AgNPs) have emerged as a groundbreaking approach to enhance the antimicrobial properties of silver, due to their large surface-area-to-volume ratio, which leads to the release of more Ag+ ions than bulk silver [45]. The enhanced antimicrobial activity of Ag+ at the nanoscale has been extensively studied in medical and healthcare applications, with AgNPs incorporated into hundreds of products, including surgical and food-handling tools, as well as catheters [46]. For instance, in the work reported by Goda et al., the in vitro release of Ag+ from the surface of AgNP-covered silicon UCs allowed the inhibition of the growth of six bacteria involved in CAUTIs [47]. Exploring novel composite materials holds promise for enhancing antimicrobial effectiveness while ensuring patient safety. Particularly noteworthy are silver nanoclusters (AuNCs), which excel in enhancing antimicrobial efficacy compared to AuNPs, thanks to their ultra-small size (1–2 nm) and adaptable properties. This marks the dawn of a new era in antimicrobial advancement, with AuNCs demonstrating their superiority in eradicating bacteria such as *E. coli* [48].

Another approach involves antibiotic-based coatings, endowed with either bactericidal or bacteriostatic properties. The predominant antibiotic compounds employed in urinary catheters are rifampin, sparfloxacin, amoxicillin, triclosan, gentamicin, and norfloxacin [49]. For instance, in a study by Fisher et al., the impregnation of urinary catheters combing rifampicin, sparfloxacin, and triclosan deterred in vitro colonization by *P. mirabilis*, *S. aureus*, and *E. coli* for 7 to 12 weeks, a longer time compared to the 1–3 days showed by commercially available antimicrobial catheters used clinically [50].

Biological fouling, or biofouling, is the intricate and unwanted accumulation of bacteria on surfaces, giving rise to biofilm growth. Since fouling is predominantly influenced by surface properties, including surface chemistry, surface energy, roughness, and wettability, modifying the surface structure offers a direct approach to controlling bacteria adhesion and infection’s consequent rise and spread [51]. For instance, anti-fouling surfaces share three common features: hydrophilicity, hydrogen bond capability, and electrically neutral capability. In this context, numerous studies revealed that the ability of hydrophilic and zwitterionic surfaces to resist fouling is linked to high hydration and surface energy. Indeed, a tightly bound water layer creates a robust physical and free energy barrier, preventing adsorption [52]. This makes it difficult for microbial biofilms to form, as the surface remains slippery and less conducive to bacterial attachment. Several polymers were employed as candidates to develop hydrophilic anti-biofouling coatings. Among them, poly(ethylene glycol) (PEG), polyethyleneimine (PEI), and zwitterion-containing polymers and their derivatives were widely investigated, since they can form hydration layers via hydrogen bonding and electrostatic attraction; hence, they are particularly effective in limiting bacterial adhesion on catheter surfaces [53,54].

A further option is based on the disruption of the bacterial biofilm [37] using hydrolytic enzymes like proteases, polysaccharide hydrolases, DNases, and bacteriophage lysins [55,56], which specifically target and break down the EPS components of the biofilm matrix. Consequently, these enzymes aid in disrupting existing biofilms formed on catheter surfaces [57]. Noteworthy in this field is the use of QS inhibitors [58]. By reducing the communication process among bacteria that coordinates biofilm formation, it is possible to disrupt their ability to adhere collectively to catheter surfaces [59]. For example, the RNAIII-inhibiting peptide is a QS inhibitor that interferes with the QS process and destroys the biofilm of *S. aureus* and *S. epidermidis* [60]. In addition, nitric oxide (NO), a lipophilic gas, can diffuse through bacterial cellular membranes, exerting nitrosative and oxidative stress. NO expresses bactericidal and biofilm disruption effects without promoting drug resistance and cytotoxicity [61].

An alternative approach is bacterial interference, in which a protective coating on the surface of UCs consists of a non-pathogenic beneficial bacterial biofilm. This method effectively deters further colonization by “bad” pathogens [25]. A distinctive aspect of this bacterial interference approach lies in the dynamic nature of the anti-pathogen coating, which is living and can renew itself to sustain its anti-pathogenic activity. For instance, Chen et al. used a probiotic biofilm consisting of *E. coli strain Nissle* 1917 to fight the spread of infections on silicone-based UCs. In this work, the surface of polydimethylsiloxane (PDMS) is first activated using CO_2_ plasma and then functionalized with mannosides to promote the adherence of *E.coli strain Nissle* 1917 [62]. The authors demonstrated the effectiveness of the bacterial interference exerted by *E. Coli Nissle* against the colonization by *Enterococcus faecalis*.

Finally, AMPs’ immobilization onto/into catheters represent one of the newest and most promising strategies to prevent biofilm formation. AMPs are short chains of aminoacidic building blocks composed of positively charged and hydrophobic residues. AMPs often exhibit a net positive charge at a physiological pH, allowing them to interact with the negatively charged microbial membranes [63]. This interaction results in the membrane’s permeabilization or disruption, causing cellular contents’ leakage and eventual cell death [39,40].

It is worth noting that in this review, the term “urinary catheters” is used to specifically refer to indwelling and intermittent urethral catheters inserted via the urethra into the bladder for urine drainage. Other types of urinary devices, such as nephrostomy tubes, ureteral stents or catheters, and urimeters, are not considered within the scope of this analysis. These devices involve distinct insertion routes, clinical indications, risk profiles, and infection mechanisms compared to urethral urinary catheters, and thus were excluded to maintain a focused and homogeneous discussion on CAUTIs.

Each of the mentioned strategies operates at distinct phases of biofilm growth, providing different effects on biofilm removal. Nevertheless, most of these strategies are still in their early stages, requiring deeper clinical evaluation to demonstrate their effectiveness. To clarify the research’s hotspots and weaknesses in the field of CAUTIs and their prevention, an in-depth investigation of the progress made so far is necessary, especially as a source of inspiration for future directions.

Along these lines, bibliometric and patent landscape analyses may provide an overview of the emerging trends in this field. Bibliometric analysis [64] provides a valuable tool for quantitatively examining the current status and trends of scientific research by considering a large volume of publications using mathematical statistics and visualization methods [65]. Analyzing publication metrics, a citation overview, and author affiliations can uncover the emerging topics, the most influential authors, and the research trends, thereby guiding strategic decision-making and resource allocation [66].

On the other hand, patent landscape analysis provides a panoramic view of technological innovation within a specific topic [67], by looking at key players, technological clusters, and commercialization pathways. Hence, patent analysis can shed light on the intellectual property landscape, highlighting the evolution of inventions, emerging technologies, and market trends [68]. Altogether, bibliometric analysis and patent landscape assessment work synergically to provide a more comprehensive understanding of the landscape of scientific progress and technological innovations [69,70,71,72].

Despite its recognized relevance, systematic bibliometric and patent analysis remains relatively underexplored. Only a few studies are currently available on patent landscapes, forecasting, and patentometry [73,74]. The proposed systematic review, based on the PRISMA methodology, focuses on bibliometric and patent landscape analyses. This study aims to map the evolution of technological innovations, identify key players, and highlight emerging strategies for preventing CAUTIs by analyzing scientific and intellectual property outputs over the past decade. In this review, we primarily focused on collecting scientific publications from 2014 to 2024, analyzing bibliometric data in terms of the number of publications per year, the most prolific authors, and countries. Similar analyses were performed for patent data, including the number of patents filed per year, most prolific applicants, countries, and top cooperative patent classification (CPC). The CPC is a worldwide spread patent classification method based on specific codes. Additionally, in both cases, a clustering analysis was performed through VOSviewer software (version 1.6.20) to develop a visual map of the co-occurrence between keywords and countries in both papers and patents. This examination assumed that papers or patents sharing similar keywords are related in some way, and these relationships can be used to identify trends in research and innovation. Based on the results outlined in this study, it will be evident that antimicrobial strategies for preventing CAUTIs represent a technological field undergoing constant evolution. To the best of our knowledge, this is one of the first systematic reviews to combine bibliometric and patent landscape analyses specifically addressing technological innovation for the prevention of CAUTIs. Our findings aim to support decision-makers, clinicians, innovation managers, and researchers by providing a comprehensive overview of the technological maturity, emerging trends, and innovation dynamics within this field. Overall, this review offers valuable insights for individuals involved in patent management, innovation strategy, research leadership, and clinical implementation, helping to guide future developments and the implementation of effective solutions for CAUTI prevention.

## 2. Materials and Methods

### 2.1. Bibliometric Database Collection and Analysis

Bibliometric data collection from scientific literature was conducted by searching the Scopus database on a specific date (15 January 2025). By applying these search criteria to the Scopus Database, the following query was obtained: (TITLE-ABS-KEY (catheter*) AND TITLE-ABS-KEY (urina*) AND TITLE-ABS-KEY (antimicrobi*)) AND PUBYEAR > 2013 AND PUBYEAR < 2025 AND (LIMIT-TO (DOCTYPE, “ar”) OR LIMIT-TO (DOCTYPE, “re”). This search retrieved 1506 documents worldwide. Scopus is a comprehensive scientific database of abstracts and citations covering many different academic disciplines. Developed by Elsevier, Scopus is designed to facilitate scholarly research, providing access to a large collection of scientific literature and citation information. Scopus offers API (application programming interface) access, allowing the integration of Scopus data for customized analyses and data retrieval. This bibliometric review follows the Preferred Reporting Items for Systematic Reviews and Meta-Analyses (PRISMA) flowchart [75]. Title, journal, all author names, month and year of publication, institution, and total number of citations were collected from each publication. Then, output analysis was carried out with Excel^®^ tools, while graphical representations of the retrieved data were obtained using Biorender. Co-authorship and co-occurrence analyses were accomplished using VOSviewer software. VOSviewer is a freely available software developed by Leiden University in the Netherlands, widely used in bibliometric studies [76,77] to identify co-author relationships, keyword co-occurrences, and co-citations, allowing the construction and visualization of bibliometric networks [78]. VOSviewer software offers three main types of maps: network visualization, overlay visualization, and density visualization. Particularly noteworthy are the network visualization and density visualization maps, which make VOSviewer an outstanding tool for mapping scientific knowledge [79]. The graphical representations of the map consist of interconnected nodes and links, where nodes symbolize specific entities such as nations, organizations, authors, and terms. The size of the nodes corresponds to their numerical frequency, while the connections between nodes denote their relationships. This visualization approach provides a clear and intuitive representation of the relationships between terms and entities within the analyzed scientific literature, facilitating the identification of key concepts, trends, and research areas.

### 2.2. Patent Database Collection and Analysis

A systematic patent search and collection was carried out employing lens.org™ (www.lens.org (accessed on 15 January 2024)), a free and open patent database and scholarly search platform that accesses Espacenet, the United States Patent and Trademark Office (USPTO), the World Intellectual Property Organization (WIPO), and Australian patent databases, providing a broad spectrum of information retrieval. On 15 January 2024, the lens.org™ database was accessed and the following search string was entered in the query text editor: (title:(catheter*) OR abstract:(catheter*) OR claim:(catheter*)) AND (title:(urina*) OR abstract:(urina*) OR claim:(urina*)) AND (title: (antimicrobi*) OR abstract: (antimicrobi*) OR claim: (antimicrobi*). The patent search was limited to patents filed between 1 January 2014 and 31 December 2024. Similarly to the bibliometric data collection, the PRISMA method was used for the patent review process. The top countries, top applicants, and classifications were examined. In addition, a network analysis based on keyword occurrence in patents was performed. Output analysis was conducted using Excel^®^ (Microsoft 365, Microsoft Corporation, Redmond, WA, USA) tools, graphical representations of the retrieved data were obtained by Biorender software (BioRender, Toronto, ON, Canada), and the network co-occurrence analysis was performed by VOS viewer software (version 1.6.20, Centre for Science and Technology Studies, Leiden University, Leiden, The Netherlands).

## 3. Results and Discussion

### 3.1. Bibliometric Data Analysis

**Figure 4:**
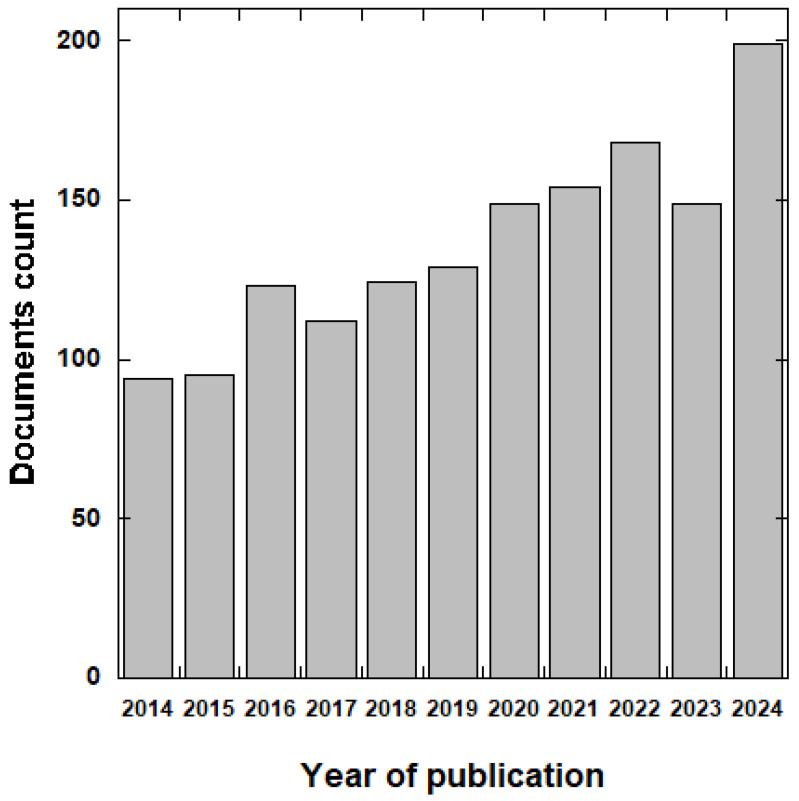
Trend of published papers over the years on CAUTIs and their prevention.

The final scoping review corpus included a total of 1506 documents. Figure 1A illustrates the flow chart based on the PRISMA method for data collection from the Scopus database. A complete analysis was conducted once all the publications were identified based on the eligibility criteria. Three keywords were used to interrogate the Scopus database: (1) catheter*; (2) urina*; (3) antimicrobi*. The initial search showed 2841 documents published from 1962 to 2024. After limiting the document types to English reviews and articles, 458 documents were removed from the database. Moreover, by restricting the research to the range between 2014 and 2024, 877 documents were deleted from the database. Table 1 and Figure 4 show the annual distribution of publications on strategies to prevent CAUTIs over the study period. The publication output expanded from 94 in 2014 to 199 in 2024. Out of a total of 1506 publications, 199 (13.21%) were recorded mainly in the year 2024, whereas 154 publications (11.42%) were published in 2022. The minimum number of publications was recorded in the early years (2014–2015).

The annual growth rate (AGR) was calculated using the formula reported by Kumar and Kaliyaperumal [80]. Table 1 reports the maximum AGR at 29.47% in 2016, followed by 29.22% in 2024. The growth in scientific production during the years 2020–2022 can surely be attributed to the global pandemic, which significantly increased hospitalizations, leading to an increase in hospital-related infections, such as urinary ones [81].

### 3.2. Predominant Journals, Highest Cited Articles, and Main Thematic Areas

Table 2 displays the top 10 journals that contribute significantly to the field of CAUTIs and their prevention. The most prolific journals are *Antibiotics* with 47 publications, followed by *BMC Infectious Diseases*, which has published 30 articles, and *Infection Control and Hospital Epidemiology* with 27 articles. The latter is also the most cited source, with 1930 citations.

Citation count is a key indicator of the importance of scientific papers, offering a quantitative measure of their impact within the scientific community [82]. For this reason, Table 3 highlights the most cited articles addressing CAUTIs and their prevention, indicating the journal and the year of publication. At the top of this list is “*Staphylococcus aureus* infections: epidemiology, pathophysiology, clinical manifestations, and management” with 2929 citations. The following is “Antimicrobial-Resistant Pathogens Associated with Healthcare-Associated Infections: Summary of Data Reported to the National Healthcare Safety Network at the Centers for Disease Control and Prevention”, with a total of 841 citations. Rounding out the top three is “Healthcare-Associated Infections, Medical Devices, and Biofilms: Risk, Tolerance, and Control” with 497 citations.

The principal thematic area significantly impacted by CAUTIs and their prevention is the medical field, comprising 1153 publications, an expected outcome as these infections are closely associated with hospitalization and the healthcare sector [93], as well as UTI-related diseases [33] and the establishment of clinical practice guidelines to manage infections [94]. Additionally, the areas of immunology and microbiology, with 275 papers, represent another closely related domain since CAUTIs are primarily caused by microbial pathogens entering the urinary tract [28,87]. Microbiological studies are crucial in identifying the pathogens responsible for CAUTIs and their underlying antibiotic susceptibility profiles [95,96,97], and understanding the mechanisms of pathogenicity [98,99]. Biochemistry, genetics, and molecular biology follow in the rankings with 205 publications. All the data about the top 10 thematic areas impacted by CAUTIs and their prevention are fully listed in Table 4.

### 3.3. Funding Sponsors and Geographical Distribution of Academic Publications

Table 5 displays the top 10 funding sponsors of academic publishers, including government agencies, research centres, and companies. The US has played a substantial role in promoting advancements in scientific research about CAUTIs and their prevention, providing most of the economic support via the National Institutes of Health (NIH) and the U.S. Department of Health and Human Services. In China, funding is provided by the National Natural Science Foundation of China (NSFC). At the same time, in the European Union, support is mostly derived from the European Regional Development Fund (ERDF) and European Commission.

**Figure 5:**
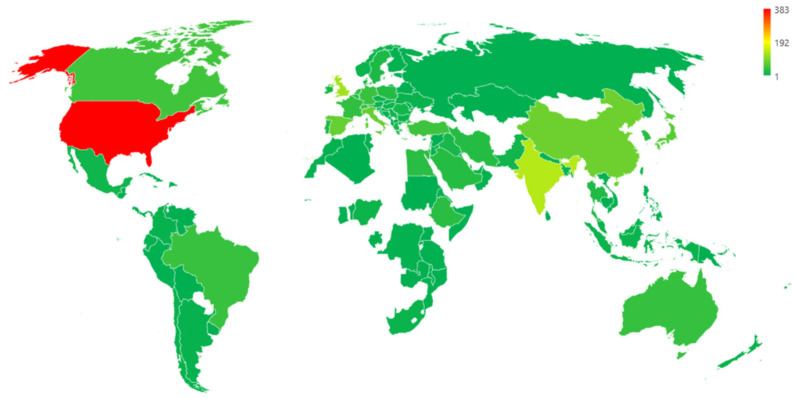
The top countries publishing papers on CAUTIs and their prevention. In red are countries with a higher number of scientific papers, and in green are the less prolific countries.

This trend is consistent among the most prolific countries. Indeed, as depicted in red in Figure 5, the United States is the most prolific world area, with 383 publications.

India and the United Kingdom have to their credit 136 and 122 publications, respectively. China follows with 82 publications and Spain with 76 articles, then Italy and Japan with 76 and 75 publications, respectively. At the end of the list, there are Canada, Turkey, and Germany with 49, 47, and 43 publications, respectively. It should be noted that the absolute number of publications is influenced by the size of the country’s population, its research funding capacity, and scientific infrastructure. Countries with significant populations and resources—such as the United States, China, and India—typically produce more scientific outputs. Therefore, comparisons without normalization by factors such as population size or research expenditure may overestimate the relative contribution of larger countries compared to smaller, yet highly research-active, nations.

### 3.4. Co-Authorship of Countries and Authors

**Figure 6:**
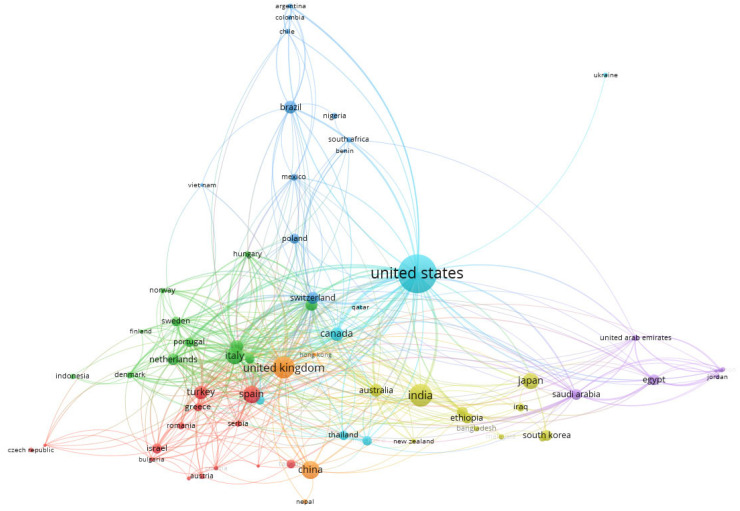
The co-authorship network of countries publishing on CAUTIs and their prevention (via VOSviewer).

Co-authorship of scientific publications among countries, reflecting the advances of researchers from different parts of the world [100], was analyzed using VOSviewer software. A bibliometric network map was generated from the analysis of the input file in VOSviewer. The graphical representations of the map consist of interconnected nodes and links. The size of the nodes corresponds to their numerical frequency, while the connections between nodes denote their relationships. The network map in Figure 6 displays the connections of the international collaborations of 115 countries on CAUTIs and their prevention.

### 3.5. Co-Occurrence Analysis of the Top Keywords in the Recovered Articles

**Figure 7:**
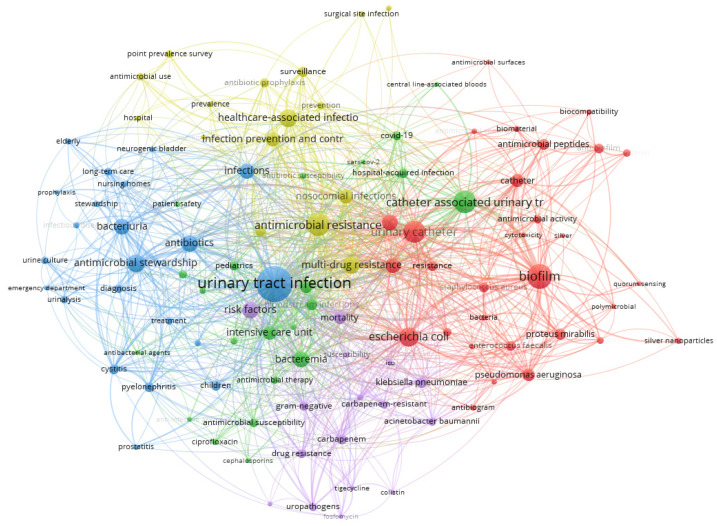
Network visualization map of the co-occurrence analysis of the keywords about strategies to prevent CAUTIs (via VOSviewer, *n* = 2813 with a minimum occurrence of keywords = 5).

A total of 1506 documents retrieved from the Scopus database were used to build the keywords network. In a scientific paper, keywords encapsulate the essence of the research and aid in indexing and categorizing articles, enhancing overall impact and detectability [101,102]. Authors strategically select keywords to align their work with specific themes, increasing the chance of the publication being read and cited. Network maps based on keywords investigate the connections among research topics and help to express their evolution over time. In this review, VOSviewer was used to extract and cluster the 2813 keywords from the 1506 retrieved publications. Figure 7 displays the network visualization map of the co-occurrence analysis of the keywords on strategies to prevent CAUTIs. Larger circles indicate a higher frequency in the co-occurrence analysis, and the color of each circle is determined by the cluster they belong to. The closer two keywords are located to each other, the stronger their relatedness. From the co-occurrence analysis of the keywords, five main clusters were obtained, displayed in red (cluster 1), green (cluster 2), blue (cluster 3), yellow (cluster 4), and violet (cluster 5). The top 50 keywords combined in the five clusters are listed in Table 6.

Cluster 1: Biofilm formation and anti-biofilm strategies

Keywords belonging to cluster 1 converge around biofilm formation and the corresponding antimicrobial strategies to manage them. This cluster displays the pathogens involved in biofilm formation, such as *Escherichia coli*, *Pseudomonas aeruginosa*, *Proteus mirabilis*, *Staphylococcus aureus*, and *Enterococcus faecalis*, contributing to persistent infections and antibiotic resistance. In this framework, Gaston et al. demonstrated that *Enterococcus faecalis* and *Proteus mirabilis* are prevalent and persistent co-colonizers on the surfaces of urinary catheters. Indeed, *E. faecalis* acts as a pioneer species on UCs, fostering persistent colonization by traditional pathogens such as *P. mirabilis*, facilitating a robust biofilm architecture, and enhancing the antimicrobial resistance for both species [103]. The use of keywords like “antimicrobial peptides” [40] and “coatings” [90], along with “silver nanoparticles” [104], stands for their efficacy in preventing biofilm formation and controlling bacterial growth. The emergence of multidrug-resistant organisms like *Methicillin-Resistant Staphylococcus aureus* (MRSA) [105] highlights the urgency of developing novel antibacterial agents and antimicrobial surfaces. Additionally, cluster 1 collects keywords about research on quorum sensing mechanisms [106] and biocompatible biomaterials that improve catheter biocompatibility [107]. Overall, this cluster reflects the multidisciplinary approach required to address the complex challenges posed by UTIs and their multifaceted nature and provides evolving strategies for their prevention and treatment.

Cluster 2: CAUTIs and their management

Cluster 2 collects keywords interconnected through their association with CAUTIs and the strategies to fight them [108], and collectively represents various aspects of infection prevention, diagnosis, and management, particularly in healthcare settings. For instance, Saini et al. developed a biomaterial modification by impregnating a silicone urinary catheter with a combination of antimicrobial substances like macrolide, azithromycin, and ciprofloxacin. The drug release profiles showed continuous release of antibiotics from catheters for about a month, leading to a long antimicrobial effect [109]. Keywords like “antibiotic susceptibility” and “antimicrobial susceptibility” shed light on the importance of assessing microbial sensitivity to specific medications to inform treatment decisions [110]. Topics such as “bacteraemia”, “CAUTIs”, and “central line-associated bloodstream infection” indicate the types of infections commonly encountered in healthcare settings [111], especially among patients in critical or intensive care units. The inclusion of “COVID-19” and “SARS-CoV-2” underscores the relevance of infectious disease management, mostly in the context of emerging infectious diseases [112].

Cluster 3: UTI diagnosis, prevention, and treatment

Cluster 3 is mainly formed by keywords interconnected through the specific types of UTIs and their antimicrobial management. Specifically, these keywords form a network representing various aspects of UTI diagnosis, treatment, and prevention. Terms like “cystitis”, “prostatitis”, and “pyelonephritis” represent specific types of UTIs, each requiring targeted treatment strategies [113,114]. Ensuring responsible management and preventative actions is crucial, especially in long-term care facilities and nursing homes, where UTIs are common [115]. Further, in an emergency scenario, prompt diagnosis and treatment of UTIs are critical to mitigate complications like bloodstream infections. Extended-spectrum β-lactamase (ESBL)-producing organisms pose challenges in UTI management, requiring careful antibiotic selection and stewardship initiatives. As described by Fujino et al. [113], acute cystitis caused by ESBL-producing *E. coli* can be treated with faropenem compounds, showing promising results.

Cluster 4: Healthcare-associated infections

Cluster 4 is related to healthcare-associated infections (HCAI), responsible antibiotic use, and antimicrobial resistance monitoring. Implementing infection prevention and control measures, such as surveillance and antibiotic stewardship programs [116,117], plays a crucial role in reducing the spread of resistant pathogens and hindering infections. Point prevalence surveys are conducted to assess the incidence of healthcare-associated infections, providing valuable data for infection control programs. In this regard, Chen et al. reported a point-prevalence survey of 52 hospitals in China and found that among 53,939 patients surveyed, the prevalence of patients with at least one HCAI was 3.7%, a lower percentage compared to the USA and Europe [118].

Cluster 5: Antibiotic resistance

Finally, Cluster 5 focuses on strategies to prevent antibiotic resistance. *Acinetobacter baumannii* and *Klebsiella pneumoniae* are well-known for their propensity to develop resistance, particularly against carbapenem antibiotics, leading to the emergence of carbapenem-resistant strains [119]. Fluoroquinolones and fosfomycin, commonly used antibiotics for treating Gram-negative bacterial infections, may encounter compromised effectiveness due to the development of drug resistance [120]. MRSA, although a Gram-positive bacterium, is also included due to its relevance in healthcare settings and its association with increased mortality [121].

**Figure 8:**
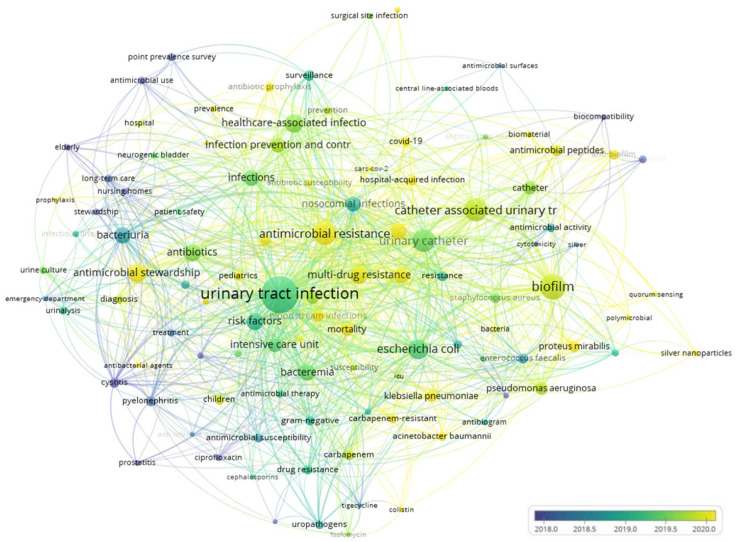
Overlay visualization map of the co-occurrence analysis of the keywords on strategies to prevent CAUTIs (via VOSviewer *n* = 2813 with a minimum occurrence of a keyword = 5).

Figure 8 illustrates the overlay visualization of the co-occurrence analysis of the top keywords, a valid tool for uncovering new trends, and mapping collaboration in the academic environment and knowledge flow. It also enables the classification of items based on the timescale. The items are color-coded according to the year of publication, with more recent terms appearing in yellow and older ones in blue. Our findings indicate that recent terms are particularly associated with antibiotic resistance and stewardship. Antimicrobial resistance poses a significant public health challenge, as drug-resistant microbes substantially contribute to morbidity and mortality in hospitals, especially in critical care units [122].

### 3.6. Patent Database Analysis

Figure 1B shows the patent review process by using the PRISMA method. The total number of recovered patents consists of 368 documents grouped into 181 families. The trends in patents related to antimicrobial strategies to prevent CAUTIs were analyzed using the retrieved The Lens database within the timeframe of 2014–2024.

**Figure 9:**
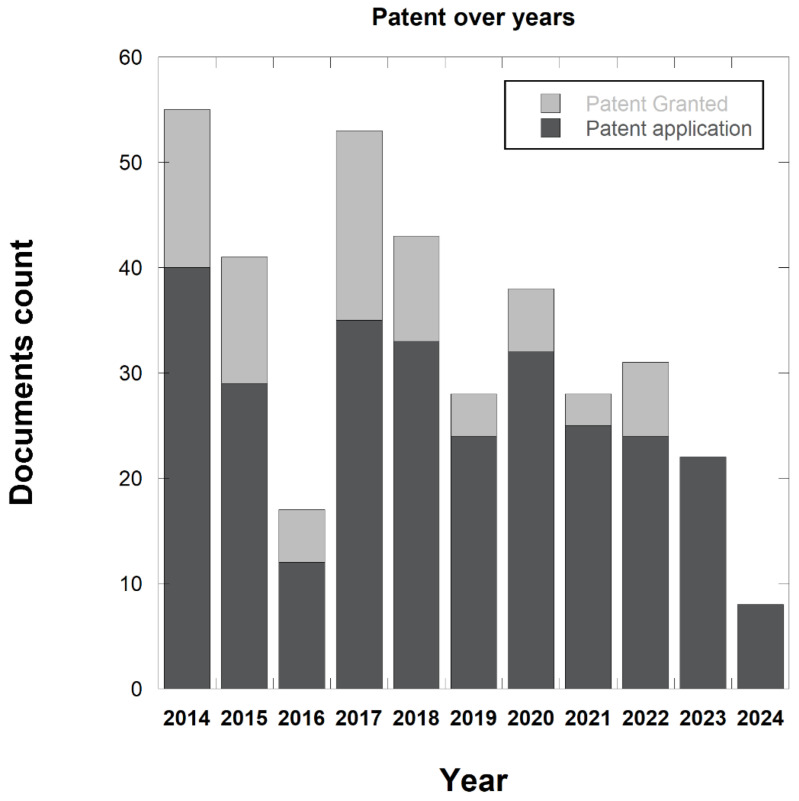
Temporal trend of patent documents, with the filed patents in dark gray and the granted patents in light gray.

Figure 9 depicts the patent trends and the document type during the mentioned period, with a percentage of 77% filed patents and 22% granted patents. An almost steady pattern of patent applications is evident over the years. However, it is worth noting that the patent publication process typically takes around 18 months from the filing date. According to this, the number of applications in the final year and a half of the analyzed period (2023–2024) might be underestimated.

### 3.7. Top 10 Patent Applicants

Recovered The Lens database identified 115 distinct applicants, with the top 10 listed in Table 7. Interestingly, except for the Swiss company Polyphor AG and the Italian Alps South, the other applicants were all from US universities and companies. In particular, the University of Texas and Polyphor AG (now SPEXIS) were the most prolific applicants in the field of CAUTIs. According to a ranking list published by the National Academy of Inventors (NAI), the University of Texas secured the third position among the top 100 US universities with granted patents in 2022. Polyphor AG is a specialist in the development of new antibiotics and drugs for the treatment of infectious, oncological, and respiratory diseases. One of the most well-known molecules developed by Polyphor is murepavadin (POL7080), a polymyxin-class antibiotic under development for severe and multidrug-resistant infections caused by Gram-negative bacteria, including *Pseudomonas aeruginosa*. From the retrieved patent database, it emerged that Polyphor AG patented peptidomimetics with antimicrobial activity, especially against Gram-negative bacteria. Bard, ranked third among applicants, is a prominent American medical device company renowned for its innovations in urology, vascular, oncology, and surgical specialty products. Tepha Medical Device, ranked fourth, specializes in the development and manufacturing of medical devices and implants made from poly-4-hydroxybutyrate (P4HB), a biomaterial derived from microbial fermentation processes. Both companies were acquired by BD (Becton, Dickinson and Company), with Bard acquired in 2017 and Tepha in 2021, further strengthening BD’s portfolio in the medical device sector.

Tracking patent citation trends can be extremely helpful in identifying which companies are engaged in active development, understanding the direction of a specific technology or field, and assessing the state of the art within a specific technological area. Patents with high citation indexes are more prone to be licensed or sold at a higher price, as they are seen as more innovative and influential in their field.

Regarding our retrieved database from The Lens, the document US 2019/0375149 A1 “Methods for 3D Printing of Poly-4-Hydroxybutyrate and Copolymers” [123], with 43 citations, was the most cited in other patents. This patent, filed in 2019 and owned by Tepha Medical Device, delineates a method to 3D-print poly-4-hydroxybutyrate (P4HB) and its copolymers. These materials, individually or in combination, can fabricate a medical implant optionally coated with a bioactive agent like antimicrobial peptides or antibiotic compounds.

In the classification of countries where patents addressing strategies to prevent CAUTIs are filed or granted, the US claims the top spot with 206 documents, followed by WIPO with 124 and Europe with 21 patents. Nevertheless, since the WIPO application is related to an undefined place for protection, these outcomes were omitted from the analysis of the potential markets for technology exploitation.

About the Cooperative Patent Classification, the retrieved results show that most of the patents were in the CPC category A61L, concerning “methods or apparatus for sterilizing materials or objects in general disinfection, sterilization or deodorization of air chemical aspects of bandages, dressings, absorbent pads or surgical articles materials for bandages, dressings, absorbent pads or surgical articles”. These CPCs adequately describe the retrieved patent database. The CPC code A61P31/04, referring to antibacterial agents, has the highest number of recorded patents with 124 documents. Next, A61L2300/404, about biocides, antimicrobial agents, and antiseptic agents, brings together 123 documents, whereas A61L29/16, indicating biologically active materials has 119 documents.

### 3.8. Co-Occurrence Analysis of the Top Keywords of the Recovered Patents

**Figure 10:**
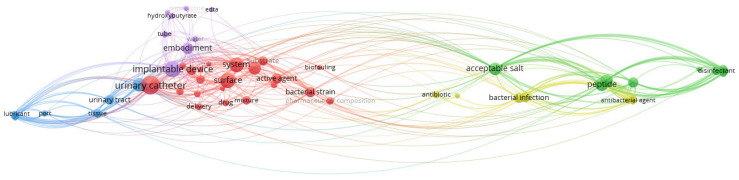
Network visualization map of the co-occurrence analysis of the patents’ keywords in strategies to prevent CAUTIs (via VOSviewer).

The correlation of keywords within the recovered patents was evaluated considering the frequency of their occurrence in both titles and abstracts. This analysis was performed with VOSviewer by selecting all the recovered patents, counting in binary mode, and including a minimum number of occurrences of a term equal to 3. The total strength of the co-occurrence links with other keywords was calculated for each keyword. Figure 10 shows the network visualization map of the co-occurrence analysis of the patents’ keywords on strategies to prevent CAUTIs. Larger circles indicate a higher frequency in the co-occurrence analysis, and the color of each circle is determined by the cluster they belong to. The closer two keywords are located to each other, the stronger their relatedness.

**Figure 11:**
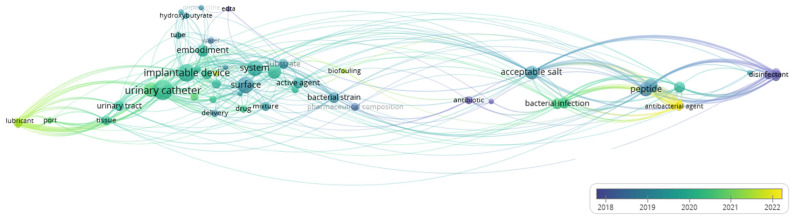
Overlay visualization map of the co-occurrence analysis of the patents’ keywords in strategies to prevent CAUTIs (via VOSviewer).

The overlay visualization, shown in Figure 11, differs from the network view in that it uses differently colored keywords. The color of a keyword refers to the patent application date which contains it. The range of colors goes from blue, representing older patents, to green and yellow, indicating newer ones. In the overlay visualization, the latest yellow-colored patents, for example, are related to the area of hydrophilic polymer-based anti-biofouling coatings.

Starting with the co-occurrence analysis of the keywords, five main clusters were obtained, displayed in red (cluster 1), green (cluster 2), blue (cluster 3), yellow (cluster 4), and violet (cluster 5). The top 40 keywords combined in the five clusters are listed in Table 8. Figure 11 shows the network visualization map of the co-occurrence analysis of the patents’ keywords in strategies to prevent CAUTIs. Larger circles indicate a higher frequency in the co-occurrence analysis, and the color of each circle is determined by the cluster they belong to. The closer two keywords are located to each other, the stronger their relatedness.

Cluster 1: Integrated antimicrobial coatings for urinary catheters

Cluster 1, in red, includes keywords related to specific technologies and approaches for targeted medical devices development to prevent and treat CAUTIs. Patents belonging to this cluster focus on urinary catheters with integrated antimicrobial properties and coatings. Indeed, keywords such as “antimicrobial coating”, “hydrophilic coating”, and “bacterial strain” suggest a reference to the coating used on the “surface” of the “urinary catheter” to inhibit microbial growth. The term “biofouling” indicates awareness about biofilm formation on device surfaces, like urinary catheters, which can contribute to urinary tract infections. Simultaneously, terms like “delivery,” “fluid,” “polymer”, and “substrate” imply the consideration of medical device design and functionality, including urinary catheters, ensuring effective fluid delivery and good biocompatibility with urinary tissue. Additionally, the mention of “bladder”, “urinary catheter”, “urinary tract infection”, and “urine” indicates specific attention to the urinary tract and associated infections.

Cluster 2: Antimicrobial formulations against uropathogen

Cluster 2, in green, is oriented toward specific microorganisms such as “Gram-negative bacteria”. Keywords like “*Escherichia coli*”, “*Acinetobacter baumannii*”, and “*Klebsiella Pneumoniae*”, focus on the specific uropathogen, whereas “antimicrobial peptide”, “peptide”, and “formulation” refer to the peptide molecules that have demonstrated their efficacy against a wide range of microorganisms, including those aforementioned. The term “disinfectant” is interconnected with substances used to destroy or inactivate microorganisms on non-living surfaces, including those in healthcare environments. Lastly, “acceptable salt” could denote salts used as excipients or stabilizers in formulations of antimicrobial or disinfectant products.

Cluster 3: Specialized coatings to enhance catheters’ functionality

Patents belonging to cluster 3, in blue, include keywords linked through their relevance to urinary tract interventions and medical device functionality. “Lubricant” suggests the use of substances to reduce friction during device insertion or movement. “Negative pressure” may relate to mechanisms involved in fluid drainage or management within the urinary tract. The concept of “protective surface area” likely relates to design features aimed at minimizing tissue damage and optimizing device functionality. “Urinary stent device” and “coating” indicate a great effort toward developing and enhancing medical devices used in the urinary tract, potentially with specialized surface coatings to enhance biocompatibility and antimicrobial properties. “Port” could refer to openings or access points in devices for fluid drainage or administration. “Urinary tract” denotes the anatomical site and biological structures involved in urinary system interventions, highlighting the importance of device design and functionality in maintaining tissue health and preventing complications.

Cluster 4: Innovative Antibacterial Treatments

Keywords in cluster 4, represented in yellow, are interconnected through their relevance to medical treatments and interventions, particularly in combating bacterial infections and diseases. “Cytotoxicity” relates to potential toxicity and harmful effects of certain substances on cells. “Parallel library” suggests the use of high-throughput screening methods for the development of novel treatments or interventions. “Therapeutic use” indicates the application of medical treatments for therapeutic purposes. “Antibacterial agent” and “antibiotic” refer to substances used to target and kill bacteria, while “phage therapy” involves the use of bacteriophages to counteract bacterial infections. “Bacterial infection” and “disease” highlight the target conditions for these treatments, emphasizing the need for effective antibacterial interventions to manage and treat infectious diseases.

Cluster 5: Implantable device to enhance efficacy and safety

Cluster 5, highlighted in violet, contains words related to medical devices, materials, and treatments. “Implantable device” and “tube” refer to medical devices designed to be placed inside the body for therapeutic or diagnostic purposes. “Embodiment” suggests different variations or forms of a particular design or concept, often referring to specific configurations or implementations of implantable devices. “P4HB” and “hydroxybutyrate” denote a biodegradable polymer used in the manufacture of medical devices. “Minocycline” is an antibiotic commonly used to prevent bacterial infection in medical devices. “EDTA” or ethylenediaminetetraacetic acid is a chelating agent used to prevent the formation of biofilms or mineral deposits on implantable devices. These terms collectively highlight different aspects of implantable device technology, materials, and treatments aimed at enhancing efficacy and safety.

Two main topics emerge from the analysis of patents resulting from the database query. The first one centers on urinary catheters with integrated antimicrobial properties, while the second one revolves around antimicrobial and antibacterial technologies development, particularly involving antimicrobial peptides and novel formulations. The first topic highlights the efforts to enhance catheter designs to prevent microbial colonization and infection, often through antimicrobial agents incorporated directly into the catheter material. Conversely, the second theme concerns the exploration of novel antimicrobial agents, formulations, and delivery methods, especially antimicrobial peptides, a promising technology with powerful antimicrobial activity against a wide range of pathogens. These findings emphasize the dual approach to tackling catheter-related infections: improving the intrinsic antimicrobial properties of catheter materials and progressing in the development of novel antimicrobial technologies with wider therapeutic use to counteract antibiotic resistance. Accordingly, Table 9 and Table 10 collecting the most cited patents referring to urinary catheters and devices with integrated antimicrobial features, report the principal antimicrobial technologies involved in the management and treatment of infectious disease, respectively. The tables include information on active patent applications, application number, application date, titles, owners, and a short description.

## 4. Conclusions

CAUTIs have emerged as one of the most widespread healthcare-associated infections, placing a substantial burden on the healthcare system and contributing to millions of cases every year. The prevention of CAUTIs is imperative to limit secondary bloodstream infections, and rates of antibiotic resistance and alleviate hospital costs. Therefore, this bibliometric and patent analysis delves into antimicrobial strategies for preventing CAUTIs, encompassing the scientific and patent landscape. Efforts have been made to minimize selection bias by using a systematic and reproducible search strategy in Scopus for bibliometric data and in The Lens for patent analysis. In addition, data extraction was conducted using predefined inclusion criteria, and visualization tools such as VOSviewer were used to ensure objectivity in identifying research trends and technological advances. Novel preventive strategies span catheter coatings, materials, and bacterial interference, leveraging nonvirulent strains to outcompete pathogens. At the same time, innovative alternatives have been developed without inducing antibiotic resistance. Nonetheless, most novel strategies lack clinical validation, necessitating further investigation. The publication output (bibliometric analysis) increased notably over the defined timeframe (2014–2024), reflecting a growing interest in addressing the challenges posed by CAUTIs. By analyzing the top cited articles and authors, the main research topics identified were antimicrobial resistance, healthcare-associated infections, and biofilm-related diseases. The geographical distribution of scholarly contributions identified the United States as the most productive country in this field. The thematic analysis revealed the multidisciplinary nature of research on CAUTIs, with medicine and immunology having the highest number of publications. This reflects the multifaceted challenges of CAUTIs and emphasizes the need for combined approaches. Furthermore, the co-authorship network of countries shows the importance of international collaboration in advancing research on CAUTIs, with the United States playing a central role. The co-occurrence analysis of keywords also unveils emerging research trends and thematic clusters, including biofilm formation, infection management strategies, antibiotic resistance, and healthcare-associated infections.

Turning to the patent database analysis in the marked period, a consistent trend in patent applications was observed over the years. The United States is the preferred place to apply for patents and a primary source of antibiofilm technology development. Trends in catheter design, featuring enhanced and integrated antimicrobial properties, and novel antimicrobial agents, particularly antimicrobial peptides, highlight ongoing efforts to counteract antibiotic resistance. From the bibliometric and patent analyses, the convergence of scientific and technological innovation reveals distinct yet complementary trends. While scientific research focuses on the design of new compounds oriented to solve the problem of multidrug resistance, patents are mostly focused on practical applications and product development. Collaboration among universities, pharmaceutical companies, and medical device manufacturers will be crucial in accelerating innovation and bringing effective CAUTIs prevention technologies to the market. Indeed, scientific reports will continue to guide technological developments, patents will acquire inspiration from prior results described in the articles, and an in-depth analysis of the technological landscape will pave the way to commercially acceptable products. In conclusion, to fight CAUTIs, researchers are directing their efforts to exploring advanced antimicrobial coatings, biofilm disruption strategies, and the development of novel antimicrobial agents. This involves supporting multidisciplinary research that integrates microbiology, materials science, and clinical studies. On the patent front, inventors and applicants are investing in innovative catheter design and antimicrobial technologies. Policies that encourage collaboration between academic institutions and industry can accelerate the development and commercialization of new products. Additionally, investing in the refinement of antimicrobial delivery systems and integrated antimicrobial features in medical devices will be crucial. By aligning funding and resources with these trends, policymakers can drive significant advancements in CAUTI prevention, ultimately improving patient outcomes and reducing healthcare costs.

## Figures and Tables

**Table 1:** Number of publications on strategies to prevent CAUTIs over the years, including annual growth rate.

Year	Number of Publications	% of Publications	AGR
2014	94	6.24	0.00
2015	95	6.31	1.06
2016	123	8.17	29.47
2017	112	7.44	−8.94
2018	125	8.30	11.61
2019	129	8.57	3.20
2020	149	9.89	15.50
2021	154	10.23	3.36
2022	172	11.42	11.69
2023	154	10.23	−10.47
2024	199	13.21	29.22

**Table 2:** Top 10 journals publishing papers on CAUTIs and their prevention.

Rank	Source	Total Publications	IF (2023)	Total Citations
1	*Antibiotics*	47	4.3	592
2	*BMC Infectious Diseases*	30	3.4	510
3	*Infection Control and Hospital Epidemiology*	27	3	1930
4	*Journal Of Hospital Infection*	25	3.9	674
5	*Infection and Drug Resistance*	23	2.9	380
6	*American Journal of Infection Control*	22	4.9	477
7	*PLOS One*	17	2.9	346
8	*European Journal of Clinical Microbiology and Infectious Diseases*	17	3.7	292
9	*Antimicrobial Resistance and Infection Control*	17	4.8	327
10	*Journal Of Infection and Chemotherapy*	15	1.9	346

**Table 3:** The highest cited articles on CAUTIs and their prevention.

Title	PY	Journal	Citations	Ref.
*Staphylococcus aureus* infections: Epidemiology, pathophysiology, clinical manifestations, and management	2015	Clinical Microbiology Reviews	3444	[83]
Antimicrobial-Resistant Pathogens Associated with Healthcare-Associated Infections: Summary of Data Reported to the National Healthcare Safety Network at the Centers for Disease Control and Prevention	2016	Infection Control and Hospital Epidemiology	962	[84]
Healthcare-Associated infections, medical devices and biofilms: Risk, tolerance and control	2015	Journal of Medical Microbiology	572	[85]
Nosocomial infections: Epidemiology, prevention, control and surveillance	2017	Asian Pacific Journal of Tropical Biomedicine	479	[86]
Tolerance and resistance of pseudomonas aeruginosa biofilms to antimicrobial agents-how *P. aeruginosa* can escape antibiotics	2019	Frontiers in Microbiology	468	[87]
Antimicrobial-resistant pathogens associated with pediatric healthcare-associated infections: Summary of data reported to the National Healthcare Safety Network	2020	Infection Control and Hospital Epidemiology	435	[88]
Clinical practice guideline for the management of asymptomatic bacteriuria: 2019 update by the Infectious Diseases Society of America	2019	Clinical Infectious Diseases	406	[89]
A review of the recent advances in antimicrobial coatings for urinary catheters	2017	Acta Biomaterialia	372	[90]
Biofilm, pathogenesis, and prevention a journey to break the wall: a review	2016	Archives of Microbiology	349	[91]
Biofilm-related disease	2018	Expert Review of Anti-Infective Therapy	328	[92]

**Table 4:** Main thematic areas on CAUTIs and their prevention impact.

Subject Area	Document Count
Medicine	1153
Immunology and Microbiology	275
Biochemistry, Genetics, and Molecular Biology	205
Pharmacology, Toxicology, and Pharmaceutics	189
Materials Science	91
Engineering	71
Chemical Engineering	52
Chemistry	50
Nursing	29
Veterinary	38

**Table 5:** Top 10 public funding sponsors in the field of CAUTIs and their prevention.

Funding Sponsor	Country	Documents Count
National Institutes of Health	United States	80
U.S. Department of Health and Human Services	United States	60
European Commission	European Union	30
European Regional Development Fund	European Union	30
National Institute of Allergy and Infectious Diseases	United States	26
National Natural Science Foundation of China	China	26
Centers for Disease Control and Prevention	United States	19
Ministerio de Economía y Competitividad	Spain	18
National Institute of Diabetes and Digestive and Kidney Diseases	United States	17
Instituto de Salud Carlos III	Spain	16

**Table 6:** The top 50 keywords (10 for each cluster) obtained by VOSviewer software from the SCOPUS database.

Keywords				
Cluster 1	Cluster 2	Cluster 3	Cluster 4	Cluster 5
biofilm	Catheter-associated urinary tract infection	urinary tract infection	antimicrobial resistance	risk factors
urinary catheter	bacteremia	bacteriuria	multi-drug resistance	mortality
*Escherichia coli*	intensive care unit	antibiotics	healthcare-associated infections	carbapenem
antimicrobials	sepsis	antimicrobial stewardship	infection prevention and control	*klebsiella pneumoniae*
antibiotic resistance	bloodstream infections	infections	nosocomial infections	drug resistance
catheter	hospital-acquired infection	cystitis	surveillance	gram-negative
*Pseudomonas aeruginosa*	pediatrics	pyelonephritis	antibiotic stewardship	uropathogens
*Proteus mirabilis*	COVID-19	diagnosis	antibiotic prophylaxis	*Acinetobacter baumannii*
*Staphylococcus aureus*	antimicrobial susceptibility	treatment	prevention	fluoroquinolones
*Enterococcus faecalis*	pathogens	children	antimicrobial use	carbapenem-resistant

**Table 7:** Top 10 applicants in strategies to prevent CAUTIs.

Applicant	% Documents	Country
University of Texas	6.80	USA
Polyphor	4.07	Switzerland
Bard	4.07	USA
Tepha medical device	3.53	USA
Hollister Inc.	2.44	USA
Akeso biomedical	2.17	USA
Griffith Donald	2.17	USA
University of Zurich	1.90	Switzerland
Alps South	1.74	Italy
University of Stanford	1.63	USA

**Table 8:** The top 40 keywords (8 for each cluster) obtained by VOSviewer software from retrieval The Lens database.

Keywords				
Cluster 1	Cluster 2	Cluster 3	Cluster 4	Cluster 5
Antimicrobial coating	Gram-negative bacteria	Urinary stent device	Cytotoxicity	Implantable device
Hydrophilic coating	*Escherichia coli*	Negative pressure	Parallel library	Tube
Bacterial strain	*Klebsiella Pneumonia*	Protective surface area	Phage therapy	Embodiment
Surface	Antimicrobial peptide	Lubricant	Therapeutic use	P4HB
Urinary catheter	Peptide	Coating	Antibacterial agent	Hydroxybutyrate
Biofouling	Formulation	Port	Bacterial infection	Minocycline
Polymer	Disinfectant	Urinary tract	Disease	EDTA
Delivery	Acceptable salt	Tissue	Antibiotic	Water

**Table 9:** List of the most relevant patents that refer to catheters with integrated antimicrobial features.

Application Number	Application Date	Title	Owners	Note	Ref.
US 2019/0168023 A1	5 December 2017	Method, System, and Devices of Safe, Antimicrobial Light-Emitting Catheters, Tubes, and Instruments	Lumen Catheters LLC	The device consists of a thin, flexible tube with an optically transparent wall. It incorporates a light transmitter, which emits UV light that is effective in killing or inactivating bacteria, viruses, and other pathogens.	[124]
US 2019/0091442 A1	30 November 2018	Coated Ureteral Catheter or Ureteral Stent and Method	Strataca Systems Limited	The device comprises a urinary catheter or stent with a protective surface area crucial for maintaining proper drainage and preventing the occlusion of drainage holes. It is coated with a specific material for lubrication, antimicrobial properties, and pH buffering.	[125]
US 9694113 B2	7 April 2015	Enhanced Pre-Wetted Intermittent Catheter with Lubricious Coating	Bard Inc.	The catheter assembly comprises a tube-like conduit. Its distal end includes at least one opening for receiving fluid from the patient’s body. The conduit and the sleeve may be arranged in a helical coil configuration. The outer surface of the conduit may feature an antimicrobial coating, to help in the inhibition of the growth of bacteria or other microorganisms on the catheter surface.	[126]
US 10709819 B2	21 September 2017	Method For Coating Catheters with a Layer of Antimicrobial Agent	Valencide LLC	A catheter made of highly flexible elastomeric material with an antimicrobial coating designed to reduce the risk of device-associated infections in the urinary tract, respiratory system, and bloodstream. This coating is intended to release iodine in a controlled manner over time, thereby inhibiting the growth of bacteria and other microorganisms on the catheter surface.	[127]
US 2022/0323787 A1	28 June 2022	Antimicrobial Light-Emitting Device and Method of Reducing Catheter-Associated Urinary Tract Infections	Lumen Catheters LLC	This invention relates to an antimicrobial urinary catheter device that uses safe, antimicrobial light to disinfect the distal urethra, urethral meatus, and the area surrounding an indwelling urinary catheter. The illumination source is designed to emit light that can eradicate pathogens without being harmful for the patient.	[128]
US 11738119 B2	8 July 2020	Antimicrobial Catheters	University Of Texas	An antimicrobial catheter made up of a low durometer aliphatic polyether polyurethane impregnated with a first antimicrobial agent (e.g., minocycline and rifampin) and coated with a second antimicrobial agent (e.g., chlorhexidine, gendine, or gardine).	[129]
US 2018/0311469 A1	27 April 2018	Antimicrobial Shield and Barrier for Urinary Catheter	Poiesis Medical LLC	A flexible protective barrier with antimicrobial, antiseptic, and antibacterial properties is designed to interact with a urinary catheter. The shield, which is adaptable, is structured to smoothly slide onto the catheter. It consists of one or more sections that are shaped to conform to and adhere to the urinary meatus and/or the surrounding skin or tissues of the targeted patient. Additionally, a flexible and expandable drape, aligned with the catheter, is connected to the shield, adding an extra layer of protection to prevent the entry of bacteria, fungi, and contaminants into the urinary tract.	[130]
US 2020/0345976 A1	08 November 2018	Drug Delivery Devices and Methods for Use with a Urinary Catheter	Taris Biomedical LLC	The urinary catheter is connected to a drug-delivery device. The flexible elongated body, along with the drug delivery lumen, is inserted into the patient’s urethra. The drug, contained within the drug chamber of the reservoir, is then delivered through the drug delivery lumen into the urinary tract.	[131]
US 2023/0321326 A1	11 April 2022	Catheter with Inherent Antimicrobial Properties	Becton Dickinson Co.	The catheter is constructed using polyhydroxyalkanoates PHA, a biopolymer known for its antimicrobial properties. PHA materials, such as poly-4-hydroxybutyrate (P4HB) and copolymers of P4HB, are particularly useful for conferring antimicrobial characteristics to the catheter.	[132]
WO 2023/215683 A1	25 April 2023	Hydrophilic Urinary Catheter Products with Microcapsules of Anti-Bacterial Agents	Hollister Inc.	The provided urinary catheter has a hydrophilic coating and incorporates anti-microbial microcapsules that release an antimicrobial agent upon irradiation.	[133]

**Table 10:** List of the most relevant patents referring to antimicrobial solutions and technologies applied in various fields.

Application Number	Application Date	Title	Owners	Note	Ref.
US 2019/0000971 A1	1 August 2016	Peptides and Antibodies for The Removal of Biofilms	Research Institute at Nationwide Children’s Hospital	Isolated or recombinant polypeptides are used in vaccinating individuals with chronic or recurrent biofilm-related diseases. The polypeptides stimulate the immune system to generate antibodies that target bacteria within the biofilm, disrupting its construction and hindering its maintenance. The antibodies generated by the immune system can prevent or clear bacterial infections by interfering with biofilm formation and function.	[134]
WO 2017/066719 A2	14 October 2016	Hu Specific Interfering Agents	Research Institute at Nationwide Children’s Hospital	The method involves the administration of interfering agents able to inhibit the binding of an HU protein to microbial DNA. HU proteins play a key role in binding DNA within the biofilm structure. The interfering agents compete with HU proteins for binding sites on microbial DNA, disrupting the stability of the biofilm.	[135]
WO 2015/181558 A1	28 May 2015	Antimicrobial Preparations, Methods for Preparing the Same and Uses Thereof to Combat Microorganisms	Ipabc Ltd.	The patent involves the preparation of antimicrobial substances containing crystalline particles of either an antimicrobial peptide or an antimicrobial polyene. The application of this purpose is to combat microorganisms. The uses of these preparations include combating a wide range of microorganisms, showcasing their versatility in antimicrobial treatments.	[136]
US 2015/0290278 A1	15 April 2015	Cationic Antimicrobial Peptides	The Hospital for Sick Children	The method combines a specific peptide and an antibiotic to treat infections synergistically. The peptide has a hydrophobic sequence (Z) with an average hydrophobicity value of at least 0.3 on the Liu-Deber scale, which contributes to the antimicrobial activity.	[137]
WO 2019/104213 A1	21 November 2018	Antibiofilm Formulations and Use Thereof	University of Texas	An approach to treat biofilm infections is addressed, presenting a comprehensive array of compositions and methods specifically tailored to address this challenging medical issue. By incorporating modified antibiotics and a diverse range of excipients, the method aims to achieve a more effective response with respect to traditional antibiotic therapies.	[138]
US 11103547 B2	2 February 2017	Methods for Disrupting Biofilms	Yissum Research Development Company of the Hebrew University of Jerusalem Ltd.	The invention involves the administration of a pharmaceutical composition to subjects in need of treatment, or for the prevention of biofilm-associated infections. The composition comprises a mixture of random-sequence peptides along with a pharmaceutically acceptable carrier.	[139]
US 2020/0138901 A1	13 January 2020	Antimicrobial Peptides, their Variants and Uses	Chain Antimicrobials Oy	Introduction of novel AMPs with a broad spectrum of action in controlling microbial growth and infections. The versatility of the peptides extends to various industries, offering solutions for microbial control and preservation in diverse settings.	[140]
WO 2022/251963 A1	2 June 2022	Polymeric Antifouling Coating with Antimicrobial Peptides	Uni of British Columbia	Compositions and methods for coating substrates with polymeric binders and AMPs, to limit biofouling and protein binding, offering solutions for reducing contamination in various applications and improving performance.	[141]
WO 2022/010942 A2	6 July 2021	Combination Therapies for the Treatment and Prevention of Biofilms	Res Inst Nationwide Childrens Hospital	A novel approach to treat and prevent biofilms and associated disorders by combining HMGB (high-mobility group box) polypeptides with anti-DNABII antibodies. HMGB polypeptides are known for their ability to disrupt biofilms, while anti-DNABII antibodies specifically target DNABII proteins, which are crucial for biofilm stability. The specific amino acid sequences in the anti-DNABII antibody ensure effective targeting and binding to DNABII proteins, enhancing the therapeutic efficacy of the composition.	[142]
WO 2021/154703 A1	26 June 2021	Compositions Including Antimicrobial Polymer-Peptide Conjugates and Uses Thereof	University of Puerto Rico	A peptide conjugate comprising a PEG arm conjugated to an AMP with a specific amino acid sequence.	[143]

## Data Availability

Data available on request.
